# The meaning of the MS Degree in Medical Physics, Part 4

**DOI:** 10.1120/jacmp.v16i2.5640

**Published:** 2015-02-19

The 2014‐2015 interview year for entry into one of the Commission on Accreditation of Medical Physics Education Programs, Inc. (CAMPEP) accredited residency programs is noteworthy for a couple of reasons. This is the first year that the majority of candidates must land a residency slot in order to qualify, eventually, to complete the American Board of Radiology certification pathway. In addition, this is the first year that a match process has been offered to the programs and candidates. According to John Antolak, Chair of Medical Physics Residency Training and Promotion Subcommittee, this year there are 295 applicants for 74 programs (but there are probably a bit more than 100 positions). Probably fewer than 70 of these are open to Master's graduates. It is clear that not all of the 74 programs are participating in the match. Considering these changes in process, I thought it could be enlightening to ask some of our interview candidates to share their experiences with us. I asked them to consider the broad question: “If you could say something directly to the academic program directors or to CAMPEP, what would it be?”

Several of the residency candidates responded to my request with a few paragraphs, which I reproduce below.

## CANDIDATE #1

The expectation cannot be to provide every applicant with a position upon graduation, but the current imbalance in the system with the acceptance of new graduate students far exceeding the placement of said students in residency positions is detrimental to the future of the program. The current competition in the residency market is astounding and will likely deter many future qualified individuals from even considering the field. Assuming the current residency allotment is valid to maintain adequate growth, then only one option needs to be considered. Restraint must be given limiting acceptance into academic programs before students are misled and riddled with even more loan debt without the prospects of directly continuing their path towards their career goals postgraduation. Program directors need to be held accountable for their placement of graduates into residencies, and should be reprimanded accordingly by you (CAMPEP) for misleading applicants in order to boost tuition or for other purposes. An incentive/ultimatum must be established in order to swiftly change the current status quo and prevent the future decline of the program. My thoughts might be ordered as follows:

1. Review current rates of placement into residency positions for postgraduate students.
2. If the rate of acceptance/placement is below a certain standard required to maintain adequate competition but far below current levels, then place the program into a type of ‘probationary’ status with regard to CAMPEP accreditation.
3. If probation occurs, have the program director correspond with the CAMPEP organization and have them discuss ways in which the organization could improve its statistics apart from lowing its acceptance rate (i.e., require more clinical training, research, etc.).
4. If the institution fails to meet the placement rate after two years of being placed on probationary status, revoke the CAMPEP accreditation from the institution.

Thanks for watching out for the students. It is greatly appreciated!

## CANDIDATE #2

I will express my thoughts and opinions on the process in this email, and I am sorry for being informal in this communication.

1. Everybody knows of the inadequate residency spots, but I believe that it is a whole lot worse than most people suggest, especially for MS students. Many openings on the AAPM website suggest that they take MS or PhD applications, but upon looking at their websites, they state they only accept PhDs. Also, many of the openings also suggest that they take MS or PhD, but upon observing past residents, they are mainly PhDs, further limiting our options. The number of applications also will increase each year, due to the fact that the applicants who did not receive a residency the previous year will reapply.
2. One of the main problems that I have with this residency is ethical issues regarding pay. Many of the larger physics groups are hiring people directly out of their residency, and to me it looks like those groups are using this process to get two years of cheap labor, because their main concern is financial. While private practice compensation is comparable to academic compensation right now, will this always be the case? If there are so many people looking for a residency position, this could cause the physics groups to reduce pay drastically, because they know that applicants need the position. I believe this raises the issues of ethics within the process.
3. To continue on about pay, some MS programs are beginning to adopt a PhD/residency program whereby, after completion of the MS program, the student spends two years in a residency and two years fulfilling requirements of a PhD. To finance this, the resident will receive the same pay as a two‐year residency, except spread out over the two‐year residency and however long it takes to finish the PhD. I believe this could raise ethical issues. Also some residencies now are beginning to offer an extra year of residency, possibly just for the cheap labor to help in the clinic. Will more institutions extend their residencies for the sake of cheap labor?
4. To talk further about pay — there are now residencies where you have to pay to complete the residency. One university is charging people $18,000 a year to attend its residency position. If there are so many people that need a residency, what is to stop every residency from following this format?

Thank you for reading this email and for allowing me to express my feelings and opinions. As a student I am concerned about this residency process, along with everybody else in this process. Students are now graduating with well over $100,000 in student loan debt, with great uncertainty about their futures.

## CANDIDATE #3

Being a medical physics master's graduate student, competing for residency positions alongside individuals with PhDs and postdocs has been a struggle. Coming from a program that graduated at least 9 master's students focusing in radiation therapy last year, it is very visible that the number of applicants far exceeds the number of therapy residency positions per year. Last year, I did not get a residency position due to individuals having greater clinical experience than I have, but I was able to locate a temporary junior physicist job. Many people I graduated with were also unable to secure a residency position and were forced to consider pursuing a PhD, which may not have been their first choice. Students receiving a master's education with the sole intention of working clinically should be able to place into residencies just as easily as PhD students who want to pursue more research, but this may not be the case in the future. I believe that the number of medical physics master's students will decrease over time with the new enforcement of a required residency to become ABR certified. Larger graduate programs in medical physics need to realize that it will become increasingly difficult for their students to secure a job after graduation, and they must decrease the number of students they are accepting per year.

## CANDIDATE #4

I would like to express my appreciation that you gave me the chance to write some of my opinions about this profession. As a master's student, I see the major problem probably is the large amount of master's applicants versus limited residency openings. This problem could be solved by either increasing the number of openings or shrinking the master's programs in the future.

However, the number of job openings may not significantly increase due to the demand unless there are big improvements in some technology which require large number of physicists. An example is that time when IGRT just came out. At this point, I would say that developing some research‐based programs which could lead to a technical revolution would potentially help solve this problem in the future. A PhD student is definitely a better candidate in such programs, compared with a master's student. Nowadays, we do have some programs that accept only PhD students, but some other programs, even though they focus on clinical work, still try to take PhD students regardless of their overqualification. This not only further narrows down the opportunities for master's students, but also wastes our education resource. Those PhD students have been trained in academic work for many years, but end up with doing clinical work which a master's graduate can do.

Therefore, why can't we explicitly classify residency programs into two types: clinical‐based (2 years clinical training +1 year following‐up clinical works), and research‐based programs (2 years clinical training +2 years research), and force clinical‐based programs to take master's students only and send all PhD candidates to research‐based programs? CAMPEP can develop a different set of accreditation requirements respecting the type of program seeking accreditation.

A possible problem might be how we could financially support those research‐based programs if they just focus on research and don't have enough money from clinical work. I would suggest that organizations such as AAPM fund them by collecting money from all other clinical‐based programs. In this way, we can use the resources from all the clinical‐based programs to support the research‐based programs and the clinical‐based programs can also benefit from the technical improvement from research‐based programs in return. This could improve the work efficiency and provide more work openings in clinics. I think in this approach, the resources in this field could be distributed more wisely.

These comments from residency candidates reveal the unexpected complexity of our education pathway. No longer do we have just the conventional and DMP financial models, but rather a confusing array of financial options is emerging. There are the ethical issues of coercing master's students into PhD programs when these programs may not have been the first choice of the student. While physics residents are usually paid similarly to medical residents, other models are starting to present, along with some corresponding questions about the ethics of compensation. Many students are already faced with $100,000 or more in debt and the option of inadequate compensation or paying for a residency experience on top of this may not be realistic. Our profession should come to a consensus about the limits of what we can expect our future colleagues to assume. How many years of training are really required? How much debt is reasonable? Who pays whom during the residency, and how much?

I submit to you that our students deserve answers to these questions. It is not right to expect them to borrow six figures for a MS degree, then fund a PhD degree and, finally, pay for a residency. Answers to these questions will determine if our profession has the character to govern itself, and also will help to define the meaning of the MS degree program in Medical Physics.

Michael D. Mills, PhD

Editor‐in‐Chief

